# Reproductive isolation in two strains of the marine rotifer *Brachionus* cf. *ibericus* (Rotifera, Monogononta) from Quintana Roo, México

**DOI:** 10.3897/BDJ.14.e128770

**Published:** 2026-04-01

**Authors:** Ailem Guadalupe Marin-Chan, Daniela Pérez-Yáñez, Gilberto Acosta-González, Jesús Alvarado-Flores

**Affiliations:** 1 Centro de Investigación Científica de Yucatán A.C. Unidad de Ciencias del Agua, Cancún, Quintana Roo, México. Calle 8, No. 39, Mz. 29, S.M. 64, CP. 77524, Cancún, México, Mexico Centro de Investigación Científica de Yucatán A.C. Unidad de Ciencias del Agua, Cancún, Quintana Roo, México. Calle 8, No. 39, Mz. 29, S.M. 64, CP. 77524 Cancún, México Mexico

**Keywords:** zooplankton, species complex, marine rotifers, reproductive isolation

## Abstract

In the Yucatán Peninsula, no reproductive analyses have been conducted on clones of the *Brachionus plicatilis* species complex, a group of significant economic, ecological and evolutionary importance. Recent studies have identified 15 distinct genetic lineages. Therefore, in this study, our aim was to cultivate and characterise clones within this group to study reproductive isolation in the Yucatán Peninsula, specifically in the State of Quintana Roo. Specimens were collected in the southeast and northwest of Quintana Roo using a Wisconsin-type zooplankton net with a 54-μm mesh size. Zooplankton samples were isolated and cultured in a bioclimatic chamber at 25°C with a photoperiod of 12:12 h of light and dark. After isolation, the rotifers were fed 1×10^6^ cells ml^-1^ of *Nanochloropsis oculata*. Subsequently, following two months of acclimatisation to laboratory conditions, we conducted taxonomic identification, created monoclonal cultures, performed morphometric characterisation, determined the population growth and resting eggs hatching percentage and performed cross-mating experiments. Twelve and eleven clones were obtained from the northwest and southeast parts of Quintana Roo, respectively. Differences were observed in the production of females, males and resting eggs, as well as in the percentage of resting eggs hatching. We determined that the southeast and northwest Quintana Roo had two different strains of rotifers due to reproductive isolation between the two regions. Therefore, we propose two populations for the coasts of Quintana Roo: Brachionus cf. ibericus "Cancún" strain and "Sian Ka'an" strain.

## Introduction

*Brachionus plicatilis* (Rotifera, Monogononta) has long been considered a cosmopolitan species and is distributed in saline aquatic environments. However, it is considered a cryptic species complex, meaning that species that are similar in morphology, physiology and biological behaviour are reproductively isolated from each other and, thus, are phylogenetically different. Moreover, these species can co-exist in the same ecological niche and are considered sibling species because of recent speciation (Gómez et al. 2002, Gómez 2005, Suatoni et al. 2006).

The first step towards recognising the diversity and complexity of *B. plicatilis* was to identify two strains with different morphological and ecological characteristics: large (L) and small (SS) morphotypes. However, the morphologies and genetic differences between the L and SS morphotypes were distinct, supporting the hypothesis that these two morphotypes should be recognised as separate species (Segers 1995). Using molecular markers and reproductive isolation experiments, Gómez et al. (1995), Serra et al. (1998) and Ortells et al. (2000) supported that *B. plicatilis* was previously recognised morphologically and ecologically as morphotype L (large strain) and *B. rotundiformis* as morphotype SS (small strain). This was confirmed by Ciros-Pérez et al. (2001b) who, based on morphological, ecological and genetic traits, reported a third morphotype SM (medium strain) in the *B. ibericus* species complex.

Numerous studies have revealed that the *B. plicatilis* species complex comprises 15 species (Mills et al. 2016). Of these, only six species have been formally described: *B. plicatilis* sensu stricto (Müller, 1786) (see Müller et al. (1786)) (morphotype L), *B. rotundiformis* Tschugunoff, 1921 (see Tschugunoff (1921)) (morphotype SS), *B. asplanchnoidis* Charin, 1947 (see Charin (1947)) (morphotype L), *B. ibericus* Ciros-Peréz, Gómez & Serra, 2001 (see Ciros-Pérez et al. (2001b)) (morphotype SM), *B. manjavacas* Fontaneto, Giordani, Melone & Serra, 2007 (see Fontaneto et al. 2007) (morphotype L), *B. koreanus* Hwang et al. 2013 (see Hwang et al. (2013)) (morphotype SM) and recently *B. paranguensis* Guerrero-Jiménez et al. 2019 (see Guerrero-Jiménez et al. (2019b)) (morphotype L). The *B. plicatilis* species complex is the most widely studied group of rotifers and has been used to investigate the evolutionary history and ecological interactions (Ciros-Pérez et al. 2001a, Ciros-Pérez et al. 2004, Montero-Pau et al. 2011 Ciros-Pérez et al. 2015, Gabaldon et al. 2015), ecotoxicology (Dahms et al. 2011), osmoregulation (Lowe et al. 2005), evolution of sexual reproduction (Carmona et al. 2009), phylogeography (Gómez et al. 2000, Gómez et al. 2007, Mills et al. 2007), aging (Snell et al. 2015) and evolutionary processes (Stelzer et al. 2011, Fontaneto et al. 2012, Tang et al. 2014).

The *B. plicatilis* species complex exhibits a high degree of genetic variability with respect to the genetic distance explained by geographical distance (Mills et al. 2016). Genetic exchange between species, as well as hybridisation between genetically different populations isolated in space, depends on dispersal, synchronised reproductive processes and established populations. Sexual reproduction is induced by environmental factors, such as food quantity and quality, population density and photoperiod. Thus, both dispersers and established populations must respond to the environment for sexual reproduction to occur, leading to the movement of genes from one species to another through interspecific hybridisation.

Stelzer and Snell (2006) demonstrated that genetically distant members of the *B. plicatilis* species complex can reciprocally induce sexual reproduction, indicating the possibility of hybridisation between sister species. The signal specificity varies amongst different species. For example, males in the *B. plicatilis* complex may respond to females of a sister species, albeit to a lesser extent than to females of the same species (Rico-Martínez and Snell 1995). Therefore, both these types of mating behaviour can act as reproductive barriers or allow hybridisation between sibling species depending on the degree of differentiation between species within the complex.

Furthermore, the *B. plicatilis* species complex in the Yucatán Peninsula has not been thoroughly investigated and essential ecological, reproductive, distribution and genetic information to interpret its complexity is lacking. Understanding the distribution of species and lineages in aquatic ecosystems is crucial for informed decision-making regarding species conservation. The Yucatán Peninsula is a region of karstic hydrogeological systems with fractures, caves and cenotes with cities of significant cultural, ecological and socio-economic importance. The city of Cancún was founded in 1974 and has experienced remarkable growth in both population and infrastructure over the past 50 years. Its territorial extent has expanded to 1,664 km², leading to significant changes in land use and ongoing impacts on aquatic ecosystems due to its expansion and the socioeconomic activities in coastal areas, mangroves and cenotes. This is further exacerbated by the millions of tourists it receives annually. In contrast, the Sian Ka'an Biosphere Reserve (Coastal Mexican Caribbean) was established as a protected area in 1986 and was added to the World Heritage list in 1987. The Reserve is dedicated to preserving a rich diversity of animal and plant species, encompassing beaches, cenotes, mangroves, tropical forests and coral reefs. Therefore, our aim was to understand the reproductive isolation and population structure of *Brachionus* spp. in the south-eastern and north-western parts of Quintana Roo, México. Our findings provide pertinent information for future genetic studies on biological connectivity and diversity conservation.

## Material and methods

**Collection of organisms**

The two sampling regions for individuals from the *B. plicatilis* species complex were chosen, based on the findings of Alvarado-Flores et al. (2021). The first region is a protected area known as the Sian Ka'an Reservation, an important eco-region for the conservation of aquatic species. The second region is Cancún, an area with a significant tourism impact that has caused the fragmentation of aquatic ecosystems, as it is immersed in an urban area. Sampling was conducted using a Wisconsin-type net with 54-μm mesh. Sampling was first conducted in Cancún (21°11'59.0" N 86°49'07.5" W) and then in an area close to the Sian Ka'an Biosphere at a site known as Pulticub (19°02'30.9" N 87°34'40.9" W). Taxonomic information reported by Ciros-Pérez et al. (2001b) was used to identify the individuals. Photographs of each organism were taken with an Imager A2 Axio ZEISS microscope using an AxioCam ICC1 camera with the AxioVision SE64ReL 4.8 Inc. 2003 software. Drawings of representative clones of the morphotypes of the Cancún and Sian Ka'an strains were made. Using the printed digital photographs and semi-transparent paper to draw the contours, they were then digitised and vectorised using Paint Windows 10 Pro Version 22H2. The specimens were identified, based on various characteristics of the lorica, including its softness, flexibility, shape and the width of the head opening. Additional distinguishing features included the number and shape of spines on the dorsal margin, the shape of the sinus and the shape and number of lobes on the dorsal margin. The number and shape of the toes, the structure of the gastric glands and the base of the body were also considered. Photographs of each organism were taken using a ZEISS Imager A2 microscope equipped with an AxioCam ICC1 camera and AxioVision SE64ReL 4.8 software. More details are provided in Supplemental material 2.

**Culture of Rotifers**

Rotifer cultures were established in a medium with a salinity of 15 g l^-1^, which was created with Red Sea® marine salt (410 ppm Ca, 1230 ppm Mg, 2.8 meq l^-1^ Alk and 7.7 dKH). The species were incubated in a bioclimatic chamber (Thermo Scientific) at 25 ± 2°C with a 12 h:12 h photoperiod of light and dark. The strains were periodically maintained by transferring the rotifers from one Petri dish to another using a 1 ml plastic Pasteur pipette and a stereoscopic microscope (Zeiss, Stemi DV4). The average number of transferred organisms was 200–250. Rotifers were fed on the alga *Nannochloropsis oculata* (Florida Aqua Farms, FL, USA). The microalgae were grown by adding F2 medium modified by Florida Aqua Farms® into a salty (15 g l^-1^) medium prepared using Red Sea® marine salt. The typical concentration of microalgae used for feeding rotifers was 1 × 10^6^ cells ml^-1^. The food density and its modifications in this study were based on experiments conducted by Rico-Martínez et al. (2013) and were guided by the experimental culture conditions outlined in the plankton culture manual by Hoff and Snell (2008). Sterile 60 x 15 mm plastic Petri dishes were used for general culturing. For population growth and reproductive experiments, polystyrene flat-bottomed 24-well plates (CostarBrand) were utilised, each with a cell growth area of 1.9 cm².

**Monoclonal culture**

After two months of acclimatisation in the bioclimatic chamber at a temperature of 25°C ± 2°C, polyclonal cultures of both strains (Cancún and Sian Ka'an) were separated into monoclonal cultures in 24-well sterile polystyrene microplates (Costar Brand). One organism was placed in each well with salted medium and microalgae as food for 4 days. Surviving organisms were then placed in Petri dishes to obtain clones of both strains. They were isolated by clone, labelled and grown under the same conditions for over 24 months.

**Morphometric analysis**

Twenty females, males and resting eggs were used from each batch of clones of both strains (Cancún and Sian Ka'an) and placed on slides for the morphometric analysis: 5 μl of Bouin (a fixative composed of picric acid, formaldehyde and acetic acid) solution and 50 μl of formalin were added to the wells containing males, females and resting eggs. Photographs were taken with an Imager A2 microscope (Axio ZEISS) using the AxioCam ICC1 with the AxioVision Inc. SE64ReL software (version 4.8). Measurements included the lengths and widths of females, males and resting eggs and the distances between the middle, central and anterior dorsal spine. A one-way permutation-based multivariate analysis of variance (PERMANOVA) was performed using a fixed factor model to determine changes between the size category of individuals by region in relation to the length and width (µm) of B. cf. ibericus. This PERMANOVA model was calculated using type III sum of squares. Statistical significance was calculated from 10,000 permutations under a reduced model. This method was carried out in PRIMER v.6.1 software. In the event that there were differences in the factor, a pairwise comparison test would be applied. The statistical analyses were based on a previous study by Arroyo-Castro (2019) on the species complex of the rotifer *Lecane bulla*. A one-way ANOVA was conducted with a 95% confidence interval (p < 0.05) using the R 3.5.1 statistical package to determine if there were statistically significant differences between measurements. If such differences existed, an analysis of the degree of similarity in variability between sites was performed using multidimensional scaling (MDS) with a Bray-Curtis distance coefficient in PRIMER v.6 software. This approach was used to identify similarities and differentiate strains.

**Intrinsic population growth experiments**

After six months of acclimatisation, population growth experiments were performed on the clones of each strain. Five organisms were placed in 2 ml in the bioclimatic chamber for 4 days in 24-well sterile polystyrene microplates (Costar®). The microalgae *N. oculata* was provided to the rotifers as food at a density of 1 × 10^6^ cells ml^-1^. The concentration of microalgae was calculated using a Neubauer chamber (Marien Field®). At the end of the experiment, the total number of females (ht), males (mt) and resting eggs (hq) were counted.

**Hatching experiments of resting eggs**

A bank of resting eggs was created from monoclonal cultures. Resting eggs were taken from the bank and stored for 15 days at 4°C in the dark to induce the hatching of eight clones of the Sian Ka'an strain and seven clones of the Cancún strain. Experiments to determine the hatching percentage were conducted by placing 20, 50 or 100 resting eggs (according to their availability) in 1 ml of salt medium in 24-well sterile polystyrene microplates (Costar®) and then in the bioclimatic chamber for 15 days. The number of organisms that hatched during the 15 days was recorded and the percentage of hatching was calculated. Each plate was checked every 12 h and the organisms that hatched were removed to avoid double counting. For the population growth experiments, a bank of resting eggs was produced and tests were conducted to induce hatching. The resting eggs produced for the experiments were collected and stored in Eppendorf tubes to re-hatch and recover clones lost during the experiments with the Sian Ka'an and Cancún strains. The resting egg bank had a total of 3367 resting eggs from the clones of the Cancún strain and 5466 from those of the Sian Ka'an strain. The percentage of hatchings after 15 days of storage in a refrigerator at 4°C is shown in Table 3.

**Cross-mating test between the clones from the Cancún and Sian Ka'an strains**

Six clones, three each from Cancún and Sian Ka'an, were used in the experiment. A total of 30 males and 15 females from each clone were selected and categorised by size (small, medium and large). The experiment involved placing the 30 males of each size category into 24-well sterile polystyrene microplates (Costar Brand), with a total volume of 1 ml per well. Each well contained 15 females that were less than 24 hours old. These experimental conditions allowed us to achieve for the testing of 18 different cross-mating combinations. The principal outcome measured was the production of resting eggs, which was assessed 48 hours after.

## Results

The clones isolated in the present work can be provisionally identified as Brachionus cf. ibericus, considering two morphologic characteristics: clones from Cancún carries inside the resting eggs in the body, while the clones of Sian Ka'an, carries outside the lorica. We obtained 12 clones from Cancún and 11 clones from Sian Ka'an. Three morphotypes were identified in each locality. Descriptions and drawings of females, males and resting eggs of some clones of strains from Cancún and Sian Ka'an were made, see Suppl. material 2. For each clone of each locality, a total of 20 adult females per clone were measured. We determined length and width. Table 1 shows all the measurements of the 12 clones from Cancún having as reference three ranges of measures that we refer to as morphotypes: small (109–113 μm), medium (120–141 μm) and large (160–163 μm). Table 1 shows all the measurements of 11 clones of Sian Ka'an having as reference three ranges of measurements that we labelled in these clones and categorised as morphotypes: small (107–135 μm), medium (143–149 μm) and large (152–159 μm). The result of the PERMANOVA showed that the length and width of the organisms varies significantly (p < 0.05, see Table 5 of Appendix 1); in addition, a pairwise comparison test was applied, which is shown in Table 6 of Suppl. material 1.

Table 2 shows the total number of females, eggs and males produced in 4 days. This was done on four clones from Cancún and eight clones from Sian Ka'an. In general, notable differences were observed between both strains. Female clones from Sian Ka'an produced more females than clones from Cancún. However, clones from Cancún produced more restings eggs than clones from Sian Ka'an. In addition, our analysis showed that the small and medium morphotypes from Sian Ka'an clones produce more females and resting eggs compared with the large morphotypes from the same locality. This observation was also seen in the Cancún clones, where the smaller and medium-sized morphotypes showed greater reproductive success than the larger morphotypes.

For instance, the Cancún clones produced more of everything. The Cancún clone C5C (median type) produced more females, while clone C18C (small type) produced more males and clone C6C (large type) produced more resting eggs. In contrast, the small clones of Sian Ka'an were the ones that produced more females (clone C12S), males (clone C12S) and resting eggs (clone C6S) as compared with other clones of the same locality.

The resting eggs of clone eight of Sian Ka'an (C8S, small morphotype) were those that presented a high percentage of hatching (75%) compared to their clones from the same study area. Clone six of Cancún (C6C, small morphotype) was the one that had a high percentage of hatching (65%) regarding the clones from the same study area. The clones with the lowest percentage of hatching were C11S (large morphotype) and C20C (large morphotype). In general, the small morphotype have resting eggs with a higher hatching percentage. However, the medium morphotype have a better uniformity of hatching percentage, on average above 50% of hatching percentage.

The Cancún strain C6C (small morphotype) has the highest hatching percentage (Table 3), the clones with the lowest hatching percentage being C18C (small morphotype) and C20C (Table 3). In the Sian Ka'an locality, the highest hatching percentage is found in the C8S (small morphotype) and C11S (large morphotype) has the lowest hatching percentage.

**Cross-mating test between clones from Cancún and Sian Ka'an**

**Figure 1.**
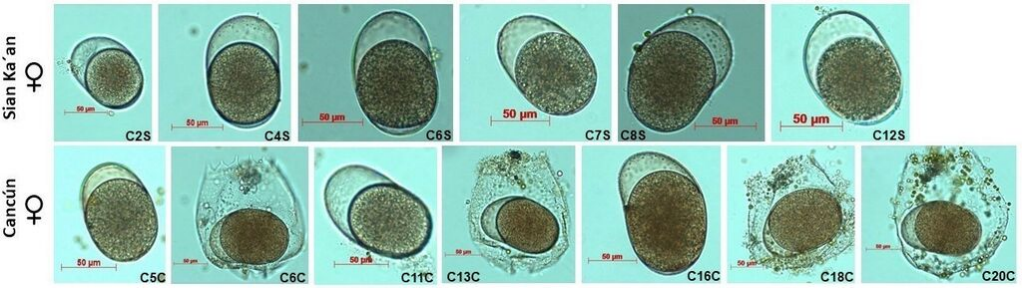
Brachionus cf. ibericus resting eggs from Quintana Roo, México.

**Figure 2.**
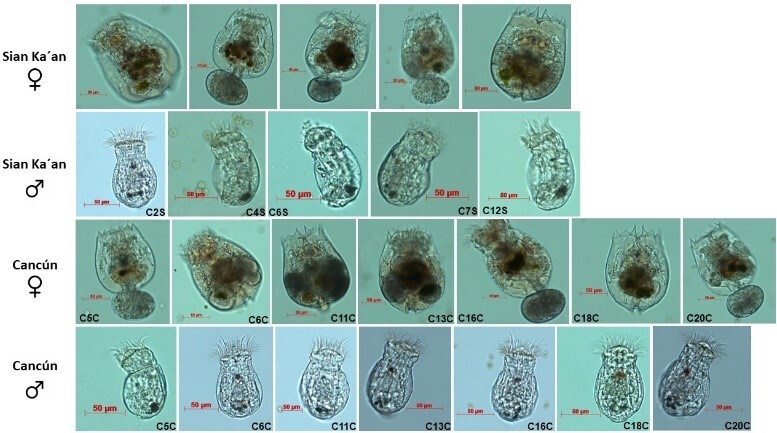
Brachionus cf. ibericus females and males from Quintana Roo, México.

Results of the cross-mating tests are shown in Table 4. From the cross-mating tests, seven combinations were fertile. In general, the males of Cancún do not fertilise the females of Sian Ka'an, while the males of Sian Ka'an do fertilise the females of Cancún. It is worth observing that all Sian Ka'an males are fertile when copulating with Cancún females. Specifically, males of the medium morphotype (C2S: the females of this clone produce resting eggs within the lorica) were able to fertilise all Cancún morphotypes (small, medium and large). However, we note that the small Sian Ka'an males do not fertilise the small Cancún females. Additionally, the large males of Sian Ka'an do not fertilise the large females of Cancún. Some forms of females, males and resting eggs were shown as morphological references in the future for identification and a morphological description in Figs 1, 2.

## Discussion

The Brachionus cf. ibericus strains from Quintana Roo, México, are part of a *B. plicatilis* species complex. This complex has three morphotypes: large (LL), medium (SM) and small (SS). Although we introduced additional morphotypes per locality, their sizes are within the measurement ranges of the SM and SS morphotypes of *B. plicatilis* (Ciros-Pérez et al. 2001b). The measurement ranges for these two morphotypes were SM = 193 μm for *B. ibericus* and SS = 148 μm for *B. rotundiformis*. In our study, the three morphotypes from Sian Ka'an were subcategorised as small (107–135 μm), medium (142–149 μm) and large (152–159 μm) and those from Cancún were small (109–113 μm), medium (120–141 μm) and large (160–163 μm). However, statistically in the pairwise comparisons, the small-C/small-S, medium-C/small-S and large-C/large-S pairs were not different, while the others were (Suppl. material 1). The morphological differentiation between strains is distinguished. Morphological, ecological, reproductive and genetic variations in the *B. plicatilis* species complex have been used to differentiate at least 15 species (Mills et al. 2016).

The results of the population growth experiment revealed differences in the number of females, males and resting eggs amongst all clones. For example, clone C5C (medium in size) from Cancún and clone C12S (small in size) from Sian Ka'an produced substantial numbers of females, males and resting eggs (Table 2) compared to other clones from the same locality. These variations in cyst, female and male production amongst clones from both sites suggest the potential co-existence of sibling species and even cross-mating between localities, influenced by the selection process through sexual reproduction.

This finding is supported by our fertilisation and cross-mating data from the clones, indicating the existence of biological connectivity in all coastal areas of Quintana Roo. Additionally, we analysed resting egg hatching and observed clear differences in the percentage of hatching amongst the clones. These differences could represent a survival strategy for populations over time, ensuring a high percentage of hatching and a substantial production of resting eggs in an unpredictable ecosystem. This high adaptability enables rotifers to be used in ecological, phylogenetic and toxicological studies.

Reproductive isolation studies in rotifers have been relatively unusual (Schröder and Walsh 2010), despite the identification of several species complexes, most of which have been examined primarily from a genetic viewpoint within the genera *Ascomorpha*, *Brachionus*, *Ephipanes*, *Keratella*, *Mytilinia*, *Platyias*, *Synchaeta*, *Euchlanis*, *Polyarthra* and *Lecane* (Arroyo-Castro 2019). The cryptic complexity in these organisms is a result of their sexual reproductive capacity and environmental adaptation, as described by Schröder and Walsh (2010). Moreover, we suggest that in karst environments and other aquatic ecosystems, hydrological connectivity is crucial in enabling encounters between sibling species of rotifers, thereby enabling potential hybridisation and gene flow mediated by sexual reproduction. Our findings specify a hybridisation process between strains of Cancún and Sian Ka´an, exhibiting distinctive morphological and reproductive traits. The most recent evidence of reproductive, morphological and genetic isolation in the karst ecosystems of the Yucatan Peninsula, Quintana Roo, México, was provided by Arroyo-Castro (2019) in the rotifer *Lecane bulla*, where two morphotypes and four clades were identified, with genetic divergence between strains ranging from 3.1% to 16%. Previously, Schröder and Walsh (2007) described three new species of *Epiphanes senta* from Chihuahua Desert (Tx, USA) and Mauna Kea (HI, USA), using integrative taxonomy (combining morphology, genetics and reproductive isolation). Therefore, gene flow mediated by sexual reproduction within rotifer species complexes is a potential aspect of differentiation and speciation over time. This highlights and enhances the importance of conserving and preserving these organisms in aquatic ecosystems, as cryptic speciation can significantly alter population structure and the dynamics of biological interactions (Jezkova et al. 2022). As a result of sexual reproduction, resistant eggs are formed, which are critical for survival and success in the environment. These egg banks represent the evolutionary history of cryptic speciation in rotifers and may constitute one of the most vital germplasm reservoirs in these ecosystems (Guerrero-Jiménez et al. 2019a; Gilbert 2019). The differences in resistant eggs between strains from Cancún and Sian Ka'an are notable, not only in their size and shape, but also in how they are carried on the body, as depicted in Suppl. material 2 and Fig. 1.

The clones from Cancún carry their resting eggs inside the body (internally), whereas those from Sian Ka'an carry them outside (externally). This distinction indicates that Sian Ka'an clones have an advantage in terms of hatching compared to the Cancún clones. As far as the authors know, when resistance eggs are retained inside the body, they gain increased protection because the lorica provides an additional layer of defence against environmental degradation and enhances resilience, facilitating dispersion through the water column by other organisms. However, this strategy has its disadvantages: the rotifer can produce only one egg at a time and if not released, the egg may eventually die. On the other hand, rotifers that release their resistant eggs externally can produce more eggs, thereby increasing their chances of survival by leaving more eggs in the sediment. However, this approach reduces the potential for dispersion, as the eggs are more likely to remain in the sediment rather than being distributed throughout the water column. Our results, as shown in Table 3, indicate that production and hatching success rates differ between strains. On average, eggs from Cancún exhibited the highest hatching success at 44.28% (n = 220), compared to those from Sian Ka'an, which had an average hatching success of 27.57% (n = 550), an increase in hatching success resulting, perhaps, due to recent speciation and hybridisation processes in strains of Cancún.

In the cross-mating tests between both strains, males from Cancún were unable to fertilise females from Sian Ka'an. Conversely, males from Sian Ka'an successfully fertilised females from Cancún. Notably, the small clone, C7S, from the Sian Ka'an locality, could only fertilise females of the large Cancún clone morphotype, C20C.

Based on the observations made in the cross-mating tests, we propose the following interpretation: Sian Ka'an males originate from a population in which females produce resting eggs outside the lorica and induce the production of females with resting eggs within the lorica when mating with females from another population, specifically Cancún. Conversely, males from a population in which females have resting eggs within the lorica cannot fertilise females from a population in which resting eggs are produced outside the lorica. This suggests reproductive isolation occurs to a certain extent, indicating recent speciation in the region. However, in future studies, whether the resting eggs produced in cross-mating tests with Sian Ka'an males and Cancún females result in a population of females having internal resting eggs must be verified in an experimental setting. This verification was not possible in the current study because of low resting egg production and the resting eggs did not hatch in the cross-mating tests.

The medium morphotype, C2S, exhibited the ability to fertilise all three Cancún morphotypes (small, medium and large). C2S generated numerous resting eggs during cross-mating with medium-sized Cancún clones.

In the Sian Ka'an clones, particularly the large morphotype C4S, fertilisation of Cancún females was challenging. C4S produced only two resting eggs in the Cancún females and one resting egg in the medium-sized females. Furthermore, C4S could not fertilise large females, indicating that male size likely influences fertilisation. The results of the cross-mating tests provided more evidence of reproductive isolation.

In general, significant morphological variation exists, especially the spines and the shape of the lorica, amongst the clones of the Cancún strain, even under identical laboratory culture conditions. All Cancún clones, except for C11C, carried resting eggs internally, a characteristic observed in *B. ibericus* by Ciros-Pérez et al. (2001b). Similar morphological variations were observed in the Sian Ka'an region. The C2S females carried the resting eggs internally, whereas the rest of the clones from the Sian Ka'an locality carried them externally. From this perspective, there may be two variants of B. cf. ibericus in Quintana Roo. These two populations might be undergoing or have already undergone speciation and there is still biological connectivity between them, indicating incomplete isolation.

This phenomenon may occur in other rotifers species, influenced by the geological and hydrological characteristics of the Yucatán Peninsula. Alvarado-Flores et al. (2021) analysed the spatial distribution of rotifer species in the Yucatán Peninsula and reported the presence of nine species of the genus *Brachionus*, three of *Keratella* and 44 of *Lecane*. Within these three genera, two rotifer morphotypes were observed: a small type in the southern region and a large type in the northern region. Arroyo-Castro et al. (2021) concluded that two different morphotypes of *L. bulla* might co-exist in the same geographical area, with one being small in size and distributed in the north-western region of Quintana Roo and the other being large in size and distributed in the south-western region.

Based on this, we proposed a second hypothesis, considering our knowledge about the distribution, morphometric analysis, reproductive isolation and demographic parameters of rotifer species. This hypothesis suggests that speciation occurs in both south-eastern and north-western Quintana Roo. These areas exhibit biological connectivity and the hydrogeological differences typical of the region likely favour speciation in rotifers and potentially other zooplankton species.

This hypothesis aligns with earlier studies on copepods in the Yucatán Peninsula (Suárez-Morales et al. 2004). They identified the southern region of the Peninsula as an ancestral zone for copepod species, which serves as a likely source of other zooplankton species found in the northern region of the Peninsula. The ancestral species spread from the southern region to the northern and speciation was facilitated over time and space. Recent studies on ostracods further support this speciation process, revealing the morphological and genetic differences in ostracod species between the northern and southern regions of the Peninsula (Macario-González et al. 2018).

Therefore, we present our opinions, based on the evidence and our results regarding the speciation of zooplankton in the Yucatán Peninsula. It is crucial to delineate and identify the biological connectivity on a regional scale, especially because the Yucatán Peninsula is affected by agriculture and tourism, which modify the ecosystem. Notably, the environmental protection areas in the Peninsula are predominantly located in the south, whereas the protection areas in the north are limited to the coastal zone. Marine speciation occurs in the northern coastal areas and involve species in the *Brachionus plicatilis* species complex and other zooplankton organisms, whereas such processes are absent within similar areas in the continent (Mercado-Salas et al. 2013).

**Figure 3.**
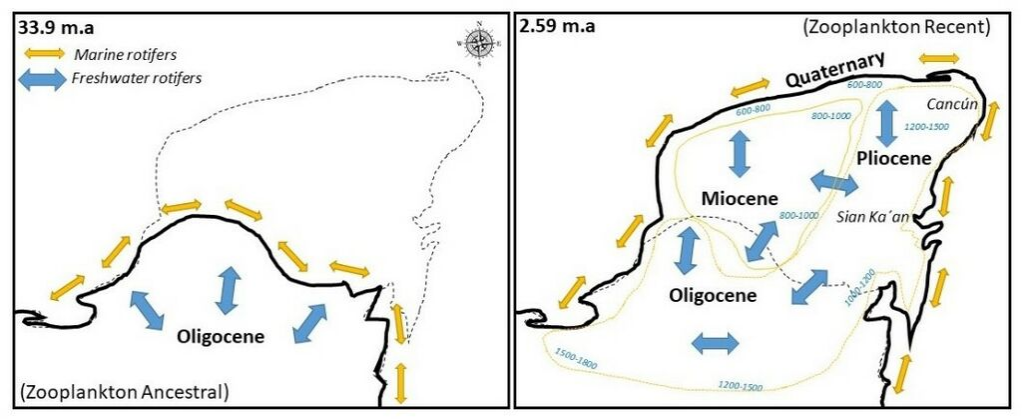
Conceptual model of reproductive isolation in zooplankton and potential zones in Yucatán Peninsula. The values in blue range from 800–1800 (mm) and correspond to annual average rainfall. m.a., mean millions of years. The two polygons with orange dotted lines correspond to the zooplankton speciation zones in the Yucatán Peninsula.

In this study, we analysed the distribution and evolution of the marine rotifer *B. plicatilis*, a species of significant commercial, genetic, toxicological and evolutionary interest. After reviewing the literature, two critical evolutionary events for zooplankton in the Yucatán Peninsula were identified. Consequently, a conceptual model was developed (Fig. 3).

The conceptual model aims to reinforce the hypothesis of the two regions of speciation in Quintana Roo, considering an additional context: the geological origin of the Yucatán Peninsula. Approximately 33 million years ago, during the Tertiary Period (Oligocene), the rotifer *Brachionus plicatilis* likely inhabited its coast. Subsequently, during the Miocene, when the Peninsula began to emerge in the north-western region, the distribution of rotifers was modified. As the Peninsula continued to grow, the dispersal patterns of rotifers were further altered, reaching the north-eastern and eastern regions of the Pliocene. Finally, during the Quaternary Period, rotifers settled in the coastal area of the Yucatán Peninsula, as it is known today. Our research hypothesis is based on the biogeographic and evolutionary analysis of copepods in the Yucatan Peninsula, as discussed by Suárez-Morales et al. (2004).

The distribution of the rotifer *B. plicatilis* is in coastal areas and the populations detected in our analysis still exhibited a certain degree of connectivity, likely indicating an evolutionary relationship with *B. plicatilis* from the Gulf of México. In studies by Rico-Martínez et al. (2013) and Moha-León et al. (2015), a strain from a coastal lagoon in the State of Veracruz was described and morphometric (length and width) and genetic analyses revealed that it has the SM morphotype (i.e., the rotifer *B. ibericus* (193.5 µm in size) and *B. almenara* (164–231 µm in size). Therefore, marine rotifer species, particularly those of the genus *Brachionus* that are distributed in the Gulf of México and the entire Yucatán Peninsula, are likely evolutionarily related. However, because of their geological periods and geographical distances, they may be undergoing speciation. Therefore, we propose two populations for the coasts of Quintana Roo: Brachionus cf. ibericus "Cancún" strain and "Sian Ka´an" strain.

## Conclusions

The rotifer population of Cancun (B. cf. ibericus strain "Cancún") carries the resting eggs internally, while the population of Sian Ka'an (B. cf. ibericus strain "Sian Ka'an") carries the resting eggs externally. Our morphometric analysis suggests that these strains are undergoing speciation, owing to the observed reproductive isolation and differences in population growth and resting egg hatching percentages. Finally, interbreeding between localities confirmed the existence of hybrids, implying that they may already be formally recognised as different species. Our study on population structure and reproductive isolation is crucial for the conservation of genetic diversity in this species complex.

## Supplementary Material

E02D2245-F3D8-5E22-81B0-9C068138910C 10.3897/BDJ.12.e128770.suppl1 Supplementary material 1 Appendice 1
Data type Statistic Manova Brief description PERMANOVA ANALYSIS (multivariate variance, based on permutations) One-way permutation-based multivariate analysis of variance (PERMANOVA), using a fixed factor model to determine changes between the size category of individuals by region about the length and width (cm) of organisms of the species B. cf. ibericus. This PERMANOVA model was calculated using the type III sum of squares. Statistical significance was calculated from 10,000 permutations under a reduced model. This method was carried out in PRIMER v.6.1 software. If there were differences in the factor, a pairwise comparison test would be applied. The results of the PERMANOVA indicated that the dimensions of the organisms, specifically their length and width, varied significantly among the analyzed size categories, with a p-value of less than 0.05, as detailed in Tables 5 and 6 of Supplementary Material 1, Appendix 1. This statistical significance suggests substantial differences in the size of the organisms when considering different categories by region. File: oo_1057525.xlsx https://binary.pensoft.net/file/1057525 Gilberto Acosta-González

D9B76FBC-E0EB-5711-A62C-430DCD356164 10.3897/BDJ.12.e128770.suppl2 Supplementary material 2 Morphological description of the Cancún and Sian Ka´an strains
Data type Morphological description Brief description Morphological description of representative clones of the study. File: oo_1110806.docx https://binary.pensoft.net/file/1110806 Ailem Guadalupe Marin-Chan, Daniela Pérez-Yañez, and Jesús Alvarado-Flores

## Figures and Tables

**Table 1.** Morphometry of the clones of the Cancún strain (C) and Sian Ka'an strain (S). The values are in micrometres.

**Morphotype**	**Clone**	**Length**	**Width**
Small	C7C	113 ± 6	100 ± 9
Small	C10C	112 ± 12	100 ± 8
Small	C12C	109 ± 12	91 ± 10
Small	C18C	111 ± 4	90 ± 3
Medium	C1C	124 ± 5	106 ± 5
Medium	C5C	130 ± 10	113 ± 7
Medium	C8C	128 ± 11	104 ± 10
Medium	C11C	120 ± 8	95 ± 8
Medium	C13C	141 ± 14	120 ± 12
Medium	C16C	123 ± 18	103 ± 14
Large	C6C	163 ± 13	119 ± 10
Large	C20C	160 ± 24	123 ± 20
Small	C6S	129 ± 18	113 ± 26
Small	C7S	122 ± 12	101 ± 11
Small	C8S	107 ± 44	91 ± 34
Small	C9S	135 ± 12	110 ± 10
Small	C10S	123 ± 17	92 ± 15
Small	C12S	128 ± 15	96 ± 21
Medium	C1S	143 ± 15	118 ± 11
Medium	C2S	145 ± 15	120 ± 9
Medium	C5S	149 ± 8	122 ± 6
Large	C4S	159 ± 17	129 ± 11
Large	C11S	152 ± 8	121 ± 11

**Table 2.** Population growth of Cancún strains (C) and Sian Ka'an (S) in females, males and resting eggs, data are presented as mean ± standard deviation.

**Morphotype**	**Clone**	**Female**	**Male**	**Resting eggs**
Small	C8S	60 ± 14.14	0 ± 0	17.5 ± 12.02
Small	C18C	54.33 ± 4.72	34 ± 7.54	1.66 ± 2.88
Small	C6S	153 ± 19.09	4 ± 5.65	49 ± 12.72
Small	C10S	87.5 ± 48.79	0 ± 0	0 ± 0
Small	C12S	173 ± 62.22	25 ± 9.89	7.5 ± 2.12
Medium	C5C	94 ± 64.21	13 ± 6.08	0 ± 0
Medium	C16C	70 ± 6.92	25.33 ± 15.27	5.33 ± 5.13
Medium	C1S	74.33 ± 14.57	0 ± 0	17 ± 1.73
Medium	C2S	71.5 ± 9.19	1.5 ± 2.12	13.5 ± 7.77
Medium	C5S	86.5 ± 19.09	0 ± 0	37 ± 32.52
Large	C6C	75.33 ± 12.01	20 ± 3.60	10.66 ± 5.85
Large	C4S	85 ± 36.76	4 ± 4	5 ± 1.41

**Table 3.** Percentage of hatching in clones of Cancún and Sian Ka'an strains, data are presented as mean ± standard deviation.

**Morphotype**	**Clone**	**Hatching** %	**Number of resting eggs**
**Small**	C18C	20 ± 0	20
**Small**	C6S	26 ± 13.41	50
**Small**	C7S	20 ± 16.32	100
**Small**	C8S	76 ± 14.29	100
**Small**	C12S	48 ± 10.32	100
**Medium**	C5C	55 ± 7.07	20
**Medium**	C11C	35 ± 14.33	100
**Medium**	C13C	60 ± 14.14	20
**Medium**	C16C	55 ± 7.07	20
**Medium**	C1S	6 ± 8.94	50
**Medium**	C2S	54 ± 6.99	100
**Medium**	C5S	0 ± 0	50
**Large**	C6C	65 ± 7.07	20
**Large**	C20C	20 ± 0	20
**Large**	C4S	15 ± 16.49	100
**Large**	C11S	2 ± 4.4	50

**Table 4.** Cross-mating experiments of the Sian Ka'an and Cancún strains.

**Morphotypes**	**Male**	**Female**	**Resting eggs**	**Fertile**
Small-Small	C18C	C7S	0	NO
Small-Small	C7S	C18C	0	NO
Medium-Small	C16C	C7S	0	NO
**Small-Medium**	**C7S**	**C16C**	**3**	**YES**
Large-Small	C20C	C7S	0	NO
**Small-Large**	**C7S**	**C20C**	**3**	**YES**
Small-Medium	C18C	C2S	0	NO
**Medium-Small**	**C2S**	**C18C**	**2**	**YES**
Medium-Small	C16C	C2S	0	NO
**Medium-Medium**	**C2S**	**C16C**	**5**	**YES**
Large-Medium	C20C	C2S	0	NO
**Medium-Large**	**C2S**	**C20C**	**2**	**YES**
Small-Large	C18C	C4S	0	NO
**Large-Small**	**C4S**	**C18C**	**2**	**YES**
Medium-Large	C16C	C4S	0	NO
**Large-Medium**	**C4S**	**C16C**	**1**	**YES**
Large-Small	C20C	C4S	0	NO
Large-Large	C4S	C20C	0	NO
