# Supplementary material for: Reproductive isolation in two strains of the marine rotifer Brachionus cf. ibericus (Rotifera, Monogononta) from Quintana Roo, México
Source: Biodivers Data J. 2026 Apr 1;14:e128770. doi: 10.3897/BDJ.14.e128770 (PMC13062783; doi:10.3897/BDJ.14.e128770)
Supplement: Supplementary material 2 — Morphological description of the Cancún and Sian Ka´an strains [file bdj-14-e128770-s002.docx]

**Morphological description of the Cancún and Sian Ka´an strains.**

**
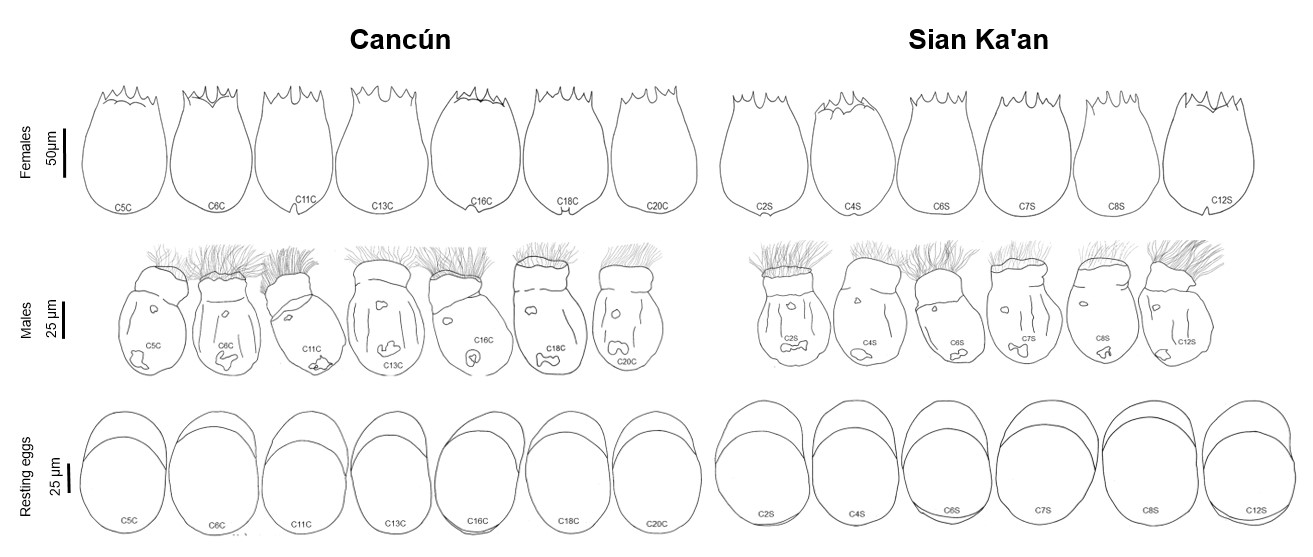
**

Figure 4. Females, males and resistant eggs of the Cancún and Sian Ka´an strains. The description of the clones is found as supplementary material.

The figure 4 shows the clones of the Cancún strain. The small morphotype is made up of C5C and C11C, both have an ovoid lorica, however, the C11C is narrower than the C5C, the middle spines of the C11C are shorter, the central spines are more prominent forming a U-shaped sinus, its lateral spines are open and pointed. In C5C the lateral spines are shorter and its spines are semi-pointed and it has a U-shaped sinus.

C16C is the only scheme with a medium morphotype, it has a semi-circular lorica, its 6 spines are similar in distance, the lateral spines are inwards, it has pointed spines, and it has a V-shaped sinus. C6C, C13C, C18C, and C20C are the large morphotype, and these clones do not differ in the lorica since all of them it is ovoid, narrow, and long, in the four clones an antero-dorsal margin with 6 pointed and triangular spines is observed, all of similar size, they have a U-shaped sinus. The medium spines are shorter than the others, their lateral spines are open.

The figure 4 also shows the 6 clones of the Sian Ka'an strain, where C2S, C4S, C6S, and C7S belong to the large morphotype, the four clones have an ovoid lorica, as for the dorsal spines all are pointed, however, C4S differs from the other 3 clones in that it has narrow and inward spines since C2S, C6S and C7S have lateral spines open outwards in the three clones the middle spines are shorter than the others except for C13C which has shorter lateral spines than the rest. They have a U-shaped sinus in the four clones of the large morphotype. The C8S, which is part of the small morphotype, has an ovoid lorica, anterior dorsal margin with 3 pairs of triangular, semi-pointed spines with a U-shaped sinus. The middle spines are similar in shape to an equilateral triangle. Finally, the C12S of the medium morphotype has a semi-circular lorica, the antero-dorsal spines are semi-pointed, with 3 pairs of spines that are similar in length and size, and it has a V-shaped sinus.

The same figure 4 shows diagrams of the males from the Cancun and Sian Ka'an strain clones. The males from the Cancun strain can reach up to L= 77.61 and A= 59.62 μm in length and in Sian Ka'an L= 72.28 and A = 51.99 μm. However, their loricas lack the ornamentation and spines as is present in females. The male lorica, like in the female, is composed of dorsal and ventral plates. It has a ciliated crown. No trophi was observed in the male. The cerebral eye is shown as well as a mark of the position of its reproductive glands. The males of the small morphtype, C5C and C11C, are not that similar, both are long and narrow, however, the male of the C5C morphotype has a semi-circular lorica, unlike C11C which has a quadrangular one, the male of C16C has a semi-circular lorica and the males of the large morphtype C6C, C13C, C18C, and C20C are similar in size, C6C and C13C have a pear-shaped lorica, and clones 18 and 20 have an ovoid one. On the right side of the figure 4, the males of the Sian Ka'an strain, C2S, C4S, C6S, and C7S, are shown. Although they are part of the same large morphotype, they have differences in the shape of the lorica. The males of the C2S and C7S clones are pear-shaped, unlike the C4S and C6S clones, which have rectangular-shaped loricas that are narrower and longer. The male of the C8S clone of the small morphotype has an ovoid, narrow, and long lorica. Finally, the male of the medium-type morph C12S, although it cannot be seen completely, seems to have a quadrangular lorica.

Finally, diagrams of the resting eggs of the Cancun and Sian Ka'an clones were made, which are shown in the figure 4 at the bottom. Few differences are observed among all the Cancun clones: the resting eggs of the small morphotypes C5C and C11C are round; C6, C13C, C18C, and C20C have narrow and elongated ovoid resting eggs, the resting egg of clone C16C, medium morphotype are similar to those of the large morphotype.

While in the clones of Sian Ka'an, in the large morphotype, C2S, C4S, C6S, and C7S there are differences in the shape despite being the same morphotype, for example, clones C2S and C6S have round resting eggs, clones C4S and C7S have ovoid resting eggs however the clone is narrower than the other clone since clone C7S is wide. Clone C8S of the small morphotype has the largest resting egg without taking into account its cover, it is ovoid shaped, finally, in the medium morphotype C12S, the shape of the resting egg is circular.
